# Characterization of unique *PMEPA1* gene splice variants (isoforms *d* and *e*) from RNA Seq profiling provides novel insights into prognostic evaluation of prostate cancer

**DOI:** 10.18632/oncotarget.27406

**Published:** 2020-01-28

**Authors:** Shashwat Sharad, Allissa Amanda Dillman, Zsófia M. Sztupinszki, Zoltan Szallasi, Inger Rosner, Jennifer Cullen, Shiv Srivastava, Alagarsamy Srinivasan, Hua Li

**Affiliations:** ^1^Center for Prostate Disease Research, Department of Surgery, Uniformed Services University of the Health Sciences and the Walter Reed National Military Medical Center, Bethesda, Maryland, 20814, USA; ^2^John P. Murtha Cancer Center, Walter Reed National Military Medical Center, Bethesda, Maryland, 20814, USA; ^3^Henry Jackson Foundation for the Advancement of Military Medicine (HJF), Bethesda, Maryland, 20817, USA; ^4^Danish Cancer Society Research Center, Copenhagen, 2100, Denmark; ^5^Computational Health Informatics Program, Boston Children’s Hospital, Harvard Medical School, Boston, Massachusetts, 02115, USA; ^6^SE-NAP Brain Metastasis Research Group, 2nd Department of Pathology, Semmelweis University, Budapest, 1085, Hungary; ^7^Urology Service, Walter Reed National Military Medical Center, Bethesda, Maryland, 20814, USA; ^*^These authors contributed equally to this work

**Keywords:** prostate cancer, PMEPA1, gene isoform, splice variant, TGF-β

## Abstract

Prostate cancer is a disease with heterogeneity of multiple gene transcriptomes and biological signaling pathways involved in tumor development. The prostate transmembrane protein, androgen induced 1 (*PMEPA1*), a multifunctional protein played critical roles in prostate tumorigenesis. The pleiotropic nature of *PMEPA1* in modulating androgen and TGF-β signaling as well as splice variants mechanisms for functional regulations of cancer-associated genes prompted us to investigate the biological roles of *PMEPA1* isoforms in prostate cancer. In addition to 4 reported *PMEPA1* isoforms (*a*, *b*, *c* and *d*), one novel isoform *PMEPA1-e* was identified with RNA Seq analysis of hormone responsive VCaP, LNCaP cells and human prostate cancer samples from The Cancer Genome Atlas (TCGA) dataset. We analyzed the structures, expressions, biological functions and clinical relevance of *PMEPA1-e* isoform and less characterized isoforms *c* and *d* in the context of prostate cancer and AR/TGF-β signaling. The expression of *PMEPA1-e* was induced by androgen and AR. In contrast, *PMEPA1-d* was responsive to TGF-β and inhibited TGF-β signaling. Both *PMEPA1-d* and *PMPEA1-e* promoted the growth of androgen independent prostate cancer cells. Although *PMEPA1-c* was responsive to TGF-β, it was found to have no impacts on cell growth and androgen/TGF-β signaling. The TCGA data analysis from 499 patients showed higher expression ratios of *PMEAP1-b* versus *-d* or *-e* strongly associated with enhanced Gleason score. Taken together, our findings first time defined the prostate tumorigenesis mediated by *PMEPA1-d* and *-e* isoforms, providing novel insights into the new strategies for prognostic evaluation and therapeutics of prostate tumor.

## INTRODUCTION

Prostate cancer is the most commonly diagnosed male malignancy and second leading cause of cancer related deaths in USA [1]. The aberrant activations of AR and TGF-β signaling executed critical functions in malignant growth of prostate and cancer metastasis [2, 3]. The precise diagnosis and prognosis of prostate cancer remained a big challenge and the identifications of novel biomarkers and therapeutic targets were constantly warranted. Our group identified *PMEPA1* gene coding for a protein of 252 amino acids (aa) (*PMEPA1-b*) in androgen treated LNCaP cells [2]. *PMEPA1* also shared homology with *C18orf1* gene mainly expressed in brain [4, 5]. Further, another transcript described as solid tumor-associated 1 protein (*STAG1*)/transmembrane prostate androgen induced protein (*TMEPA1*) with 287 aa (*PMEPA1-a*) in renal cell carcinoma [6]. These findings were followed by the discoveries of additional two isoforms in colon and lung cancers, coding for 237 aa (*PMEPA1-c*) [7] and 259 aa (*PMEPA1-d*), respectively [8]. Further, the *PMEPA1* gene locus was denoted on the human chromosome 20, absolute position 56286592-56234606. The first reported *PMEPA1* gene isoform (*PMEPA1-b*) was defined as an androgen inducible, and all other isoforms including *PMEPA1-a* (287 aa), *PMEPA1-c* (237 aa) and *PMEPA1-d* (259 aa) were found in non-androgenic cellular contexts [1, 6–10].

Our previous study defined the direct AR binding sites within the promoter of *PMEPA1* gene by GREF_GATA model [11]. Additionally, the androgen responsive PMEPA1 protein negatively regulated the protein level of AR through a feedback loop by recruiting E3 ubiquitin ligase NEDD4 [12, 13]. Quantitative-PCR (Q-PCR) analysis in matched prostate normal/tumor tissues showed that decreased expression of *PMEPA1* in approximately 65% of prostate tumors, which also strongly associated with higher pathologic stage and serum prostate-specific antigen (PSA) [13]. It was shown that the methylation of *PMEPA1* gene promoter accounted for the silencing of *PMEPA1* in prostate cancer cells *in vitro* and *in vivo* [14, 15]. The *PMEPA1* silencing conferred the development of resistance to AR inhibitors *in vitro*, as well as promoted the androgen independent xenograft growth of prostate tumor in nude mice [16]. *PMEPA1* was also reported as a TGF-β regulated gene in context of both prostate cancer and non-prostate solid tumors including colon, lung and breast cancers [7, 8, 10]. Subsequent studies indicated that *PMEPA1* participated in a negative feedback loop to control TGF-β/SMAD signaling [17–20]. Our earlier study revealed that *PMEPA1* inhibited the growth of both hormone dependent and independent prostate cancer cells [12, 13]. In contrast, *PMEPA1* was also reported to promote the proliferation of AR negative PC3 cells by suppressing p21 expression through a negative feedback loop with TGF-β [21]. Further, a recent study showed that the loss of membrane-anchored PMEPA1 protein facilitated metastasis of prostate cancer via activating TGF-β signaling by sequestering SMAD2/3 in proteasome independent way [3].

Cumulatively, these findings underscored the multi-function features of *PMEPA1* gene, and further suggested its expressions and biological functions were dependent on the cellular context centering androgen and TGF-β signaling. The alternative splicing variant mechanism had also been shown to be important for diversifying functions of tumor-associated genes. The RNA splicing mechanism across the tumors allowed the expressions of multiple RNA and protein isoforms from one gene, serving as a major contributor to diversities of transcriptomes and proteomes [22, 23]. The previous studies had implied splicing variants mechanism accounted for the formation of *PMEPA1* gene isoforms and its multi-functional features in tumorigenesis. Further, earlier studies from our and other groups explored *PMEAP1* gene isoforms (*a* and *b*) in the initiation and development of prostate tumors via interrupting AR and/or TGF-β signaling. Here, we focused on defining the expressions, regulations and biological behaviors/functions of understudied *PMEPA1* isoforms (*d* and *e*) in the context of both androgen and TGF-β signaling, and further exploration of the clinical significances and relevance of these isoforms in prostate tumor.

## RESULTS

### Structures and expressions of PMEPA1 isoforms (c, d and e) in prostate cancer cells

**Figure 1:**
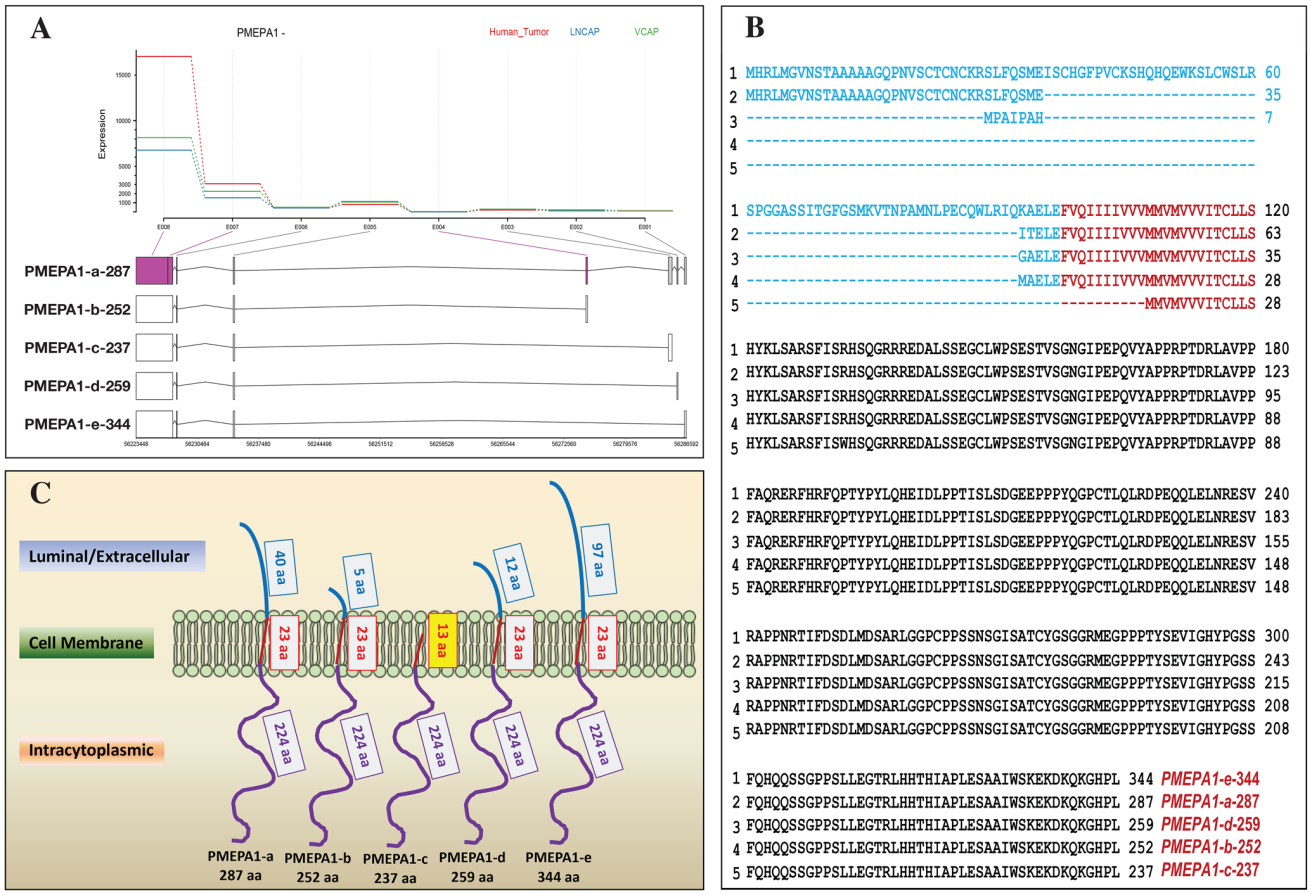
Identifications and expressions of *PMEPA1* isoforms in prostate cells. (**A**) Analysis of RNA Seq data from LNCaP, VCaP prostate cancer cell lines and TCGA human prostate tumors for *PMEPA1* gene isoforms. Structures of *PMEPA1* gene and mRNA of five isoforms were shown. As the program used for analysis was preset, RNA Seq data was presented in 3′ – 5′ orientation. The vertical bars and rectangles represented exons and UTR, respectively. (**B**) Alignment of the predicted amino acid (aa) sequences of PMEPA1 isoforms. Three function domains were predicted with a type 1b membrane protein: N-terminal (luminal/extracellular) (blue), membrane spanning (red) and cytoplasmic (black). (**C**) The sketch of protein structures of PMEPA1 isoforms including luminal/extracellular domain (blue), transmembrane domain (red) and intracellular domain (purple).

RNA-Sequencing approach was utilized to analyze *PMEPA1* gene splice variants and their relative expression ratios in hormone responsive prostate cancer cells (LNCaP and VCaP cell lines) as well as The Cancer Genome Atlas (TCGA) dataset comprising of 130 malignant and 55 benign human prostate samples (https://portal.gdc.cancer.gov/projects/TCGA-PRAD v10.0). In addition to the most abundant isoforms *PMEPA1-a* and *PMEPA1-b*, isoforms *PMEPA1-c* (open reading frame (ORF) 237 aa), *PMEPA1-d* (ORF 259 aa) with lower level of expression were also detected (Table 1). A novel unreported isoform with an ORF of 344 aa was identified and designated as *PMEPA1*-*e* in accordance with the current nomenclature of reported *PMEPA1* isoforms (*PMEPA1-a*, *-b*, *-c* and *-d*) (Figure 1A and Table 1). As PMEPA1 was predicted to be a type 1b membrane protein, three domains designated as N-terminus (luminal), membrane-spanning and C-terminus cytoplasmic domains were shown in PMEPA1 protein (Figure 1C). The predicted amino acid sequence alignment showed striking variations at the N-terminus and a high homology at the C-terminus of the PMEPA1 isoforms (Figure 1B). The N-terminus of PMEPA1-a, -b and -d isoforms contained 40 aa, 5 aa and 12 aa, respectively. Interestingly, the newly discovered PMEPA1-e isoform contained the longest N-terminus of 97 aa. Additionally, PMEPA1 isoforms a, b, d and e shared a highly conserved membrane spanning domain of 23 aa. In contrast, PMEPA1-c isoform lacked N-terminus luminal domain and contained a truncated membrane spanning domain of 13 aa. A conserved intracytoplasmic domain of 224 aa was detected in all PMEPA1 isoforms (Figure 1B and 1C).

**Table 1:** *PMEPA1* isoforms assessed in the RNA Seq dataset of TCGA-PRAD v10.0

PMEPA1 Isoforms	Open Reading Frame	Ref Seq	Consensus Coding Sequence	Tumor (*N* = 499) Expression Mean Log2 TPM
PMEPA1-c	237 amino acid	NM_199171.2	CCDS13464	4.848
PMEPA1-d	259 amino acid	NM_001255976.1	None	0.28735
PMEPA1-e	344 amino acid	ENST00000395819.3	None (intron retention)	1.4356

### Analysis of exon-intron structures of PMEPA1 isoforms

**Figure 2:**
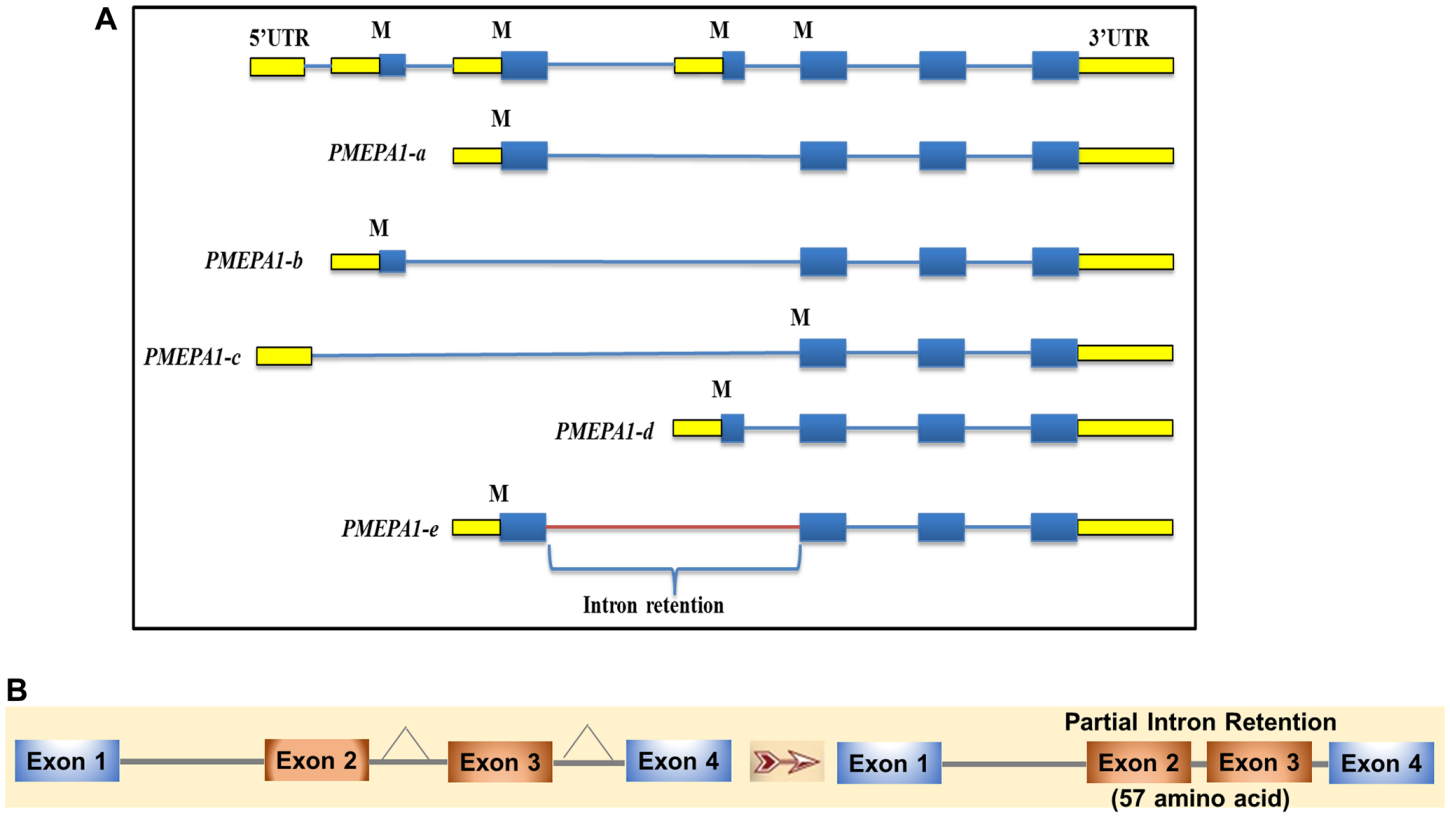
*PMEPA1* splice variants (isoforms) exon-intron structure. (**A**) Genome schematic representation of *PMEPA1* isoforms. The panel indicated the structures of *PMEPA1* isoforms and respective intron-exon corresponding to *PMEPA1* gene. (**B**) Schematic representation of partial intron retention of *PMEPA1-e* isoform. The panel indicated the intron was retained between exon 2 and 3 of *PMEPA1-e* isoform.

The database searches showed that *PMEPA1* gene sequence was within the genomic clones designated as RP5-1059L7 (AL21913), RP4-718J7 (A1035541) and RP5-1007E6 (AL161943). *PMEPA1* gene was located on chromosome 20q13.2–q13.33 between D20S183 and D20S173 micro satellite marker 1, 3 and 4 with approximately 62 kb in length and the coding region containing 6 exons and 5 introns. The exon-intron structures of the isoforms were presented in Figure 2A. *PMEPA1* isoform *a*, *b* and *d* contained 4 exons and 3 introns, whereas isoform *c* contained 3 exons and 2 introns. Isoform *e* contained 4 exons and 3 introns. A close inspection of RNA Seq data of *PMEPA1-e* isoform further revealed an additional 57 aa in comparison to *PMEPA1*-*a* which may be due to a partial intron retention between exon 2 and 4 maintaining the open reading frame (Figure 2B). As a result, the amino acids from residue 37 to 93 were unique to PMEPA1-e (Figure 2B). The translation initiation site of *PMEPA1*-*e* remained the same as *PMEPA1*-*a*, and the amino acid sequences were identical up to 36 residues.

### Distinct regulations of expressions of PMPEA1 isoforms (d and e) by androgen or TGF-β in prostate cancer cells

To explore the transcript levels of *PMEPA1* isoforms (*c*, *d* and *e*) in prostate cancer cells, isoform specific primers were designed to differentiate each isoform by their unique 5′ sequences transcribed. The mRNA levels of *PMEPA1* isoforms were analyzed by quantitative RT-PCR in androgen dependent LNCaP, VCaP, LAPC4 cells and TGF-β responsive DU145 and PC3 cells. The transcript levels of *PMEPA1-c* and *PMEPA1-d* were detected in both androgen and TGF-β responsive prostate cancer cells. In contrast, the mRNA of *PMEPA1-e* was only detectable in AR positive VCaP and LAPC4 cells (Table 2). Despite its presence by RNA Seq data, LNCaP cells failed to yield positive result for detecting *PMEPA1-e* isoform mRNA which may be due to low level.

**Table 2:** Relative expression ratio of *PMEPA1* isoforms in prostate cell lines detected by Q-PCR

Cell Line	Signaling	*PMEPA1-c*	*PMEPA1-d*	*PMEPA1-e*
**LNCaP**	Androgen sensitive	1	0.13	No
**VCaP**	Androgen sensitive	1	0.02	Yes
**LAPC4**	Androgen sensitive	1	0.01	Yes
**DU145**	AR (–) TGF-β signaling (+)	1	0.80	None
**PC3**	AR (–) TGF-β signaling (+)	1	0.76	None

**Figure 3:**
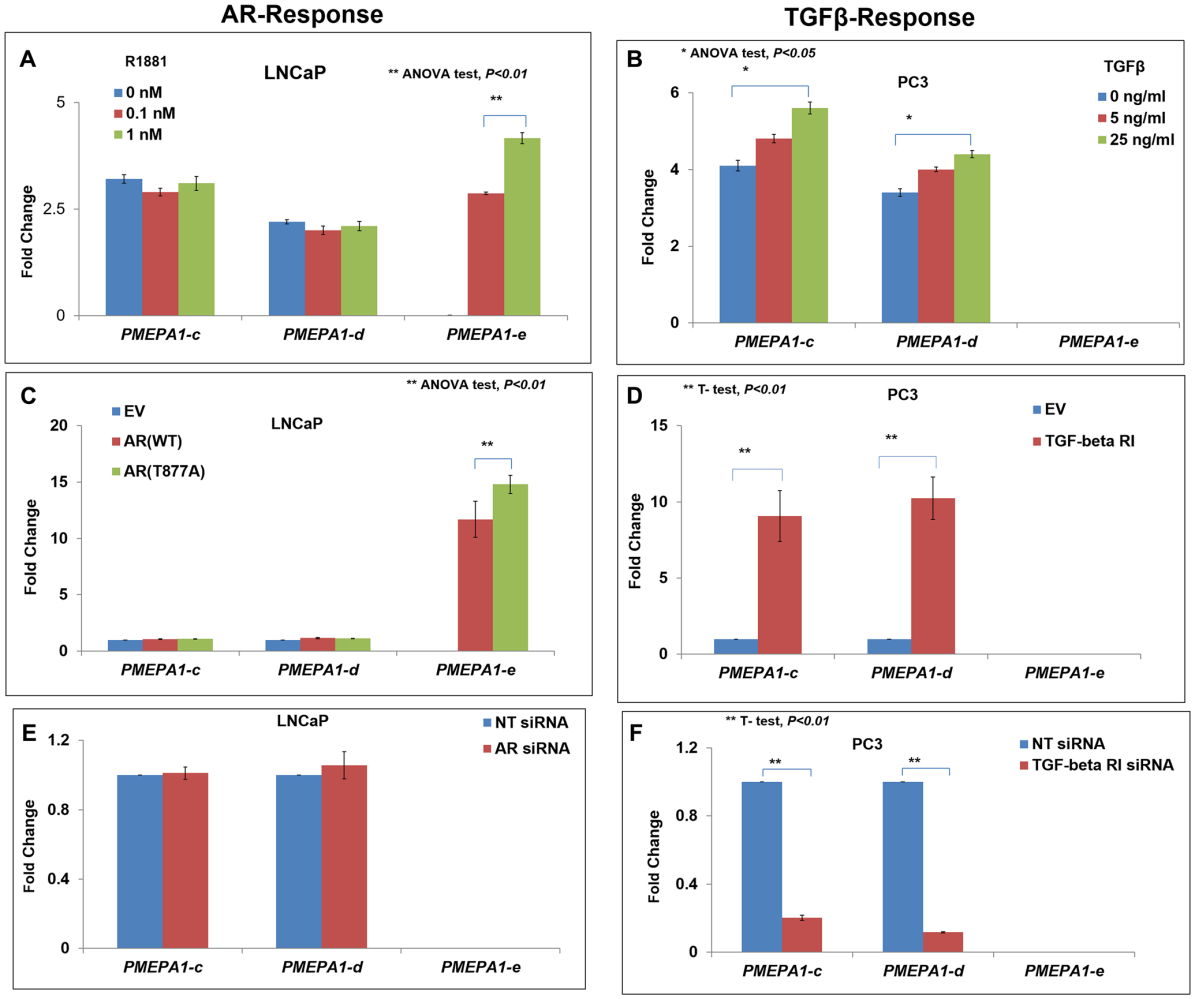
The *PMEPA1* isoforms (*c*, *d* and *e*) were categorized into two subgroups in prostate cancer cells: androgen responsive and TGF-β responsive. The transcript levels of *PMEPA1* isoforms (*c*, *d* and *e*) were assessed by Q-PCR in androgen responsive LNCaP cells and TGF-β responsive PC3 cells. (**A**) LNCaP cells treated with R1881 at linear dosages of 0, 0.1 and 1.0 nM for 24 hours. (**B**) PC3 cells treated with TGF-β at linear dosages of 0, 5 and 25 ng/ml for 24 hours. (**C**) LNCaP Cells were transfected with pCMV-XL5-wild-type AR, -T877A mutant AR and pCMV-XL5 as control. (**D**) LNCaP Cells were transfected with AR siRNA and scramble siRNA as control. (**E**) PC3 cells were transfected with pcDNA3.1-*TGFRI* and pcDNA3.1 as control. (**F**) PC3 cells were transfected with *TGFRI* siRNA and non-target siRNA as control.

*PMEPA1-a* was reported as TGF-β inducible isoform whereas *PMEPA1-b* had been identified as an androgen responsive isoform with prostate abundance [2, 12, 13]. However, the responsiveness of *PMEPA1-c, d* and *e* isoforms to androgen or TGF-β were still unclear. The transcript level of *PMEPA1-e* showed dose-dependent increases in response to androgen treatment in LNCaP cells, consistent with its restricted expression pattern in androgen dependent prostate cancer cells. On the contrary, no responses to synthetic hormone R1881 treatment were detected for *PMEPA1* isoforms *c* and *d* in LNCaP cells (Figure 3A). Both of *PMEPA1* isoforms *c* and *d* were up-regulated by TGF-β in PC3 cells. Whereas, the transcript level of *PMEPA1-e* was not induced by TGF-β treatment (Figure 3B). As expected, heterologous expression of wild-type and T877A mutant AR induced the expression of *PMEPA1-e* in LNCaP cells. However, ectopic AR had no impact on the transcript levels of isoform *c* and *d* (Figure 3C). Similarly, ectopic TGF-β receptor I (*TGFRI*) enhanced the mRNA levels of *PMEPA1-c* and *-d* in PC3 cells (Figure 3D). Additionally, knockdown of AR had no effect on the transcript levels of isoform *c*, *d* and *e* in LNCaP cells (Figure 3E). Consistently, silencing of *TGFRI* led to down-regulation of transcript levels of isoforms *c* and *d* in PC3 cells (Figure 3F). The transcript level of *PMEPA1-e* was also not impacted by ectopic *TGFRI* (Figure 3D). Taken together, our findings rendered *PMEPA1-e* as androgen responsive and *PMEPA1-c* and *PMEPA1-d* as TGF-β responsive in prostate cancer cells.

### PMEPA1 isoform d and e promoted the growth of TGF-β responsive prostate cancer cells

**Figure 4:**
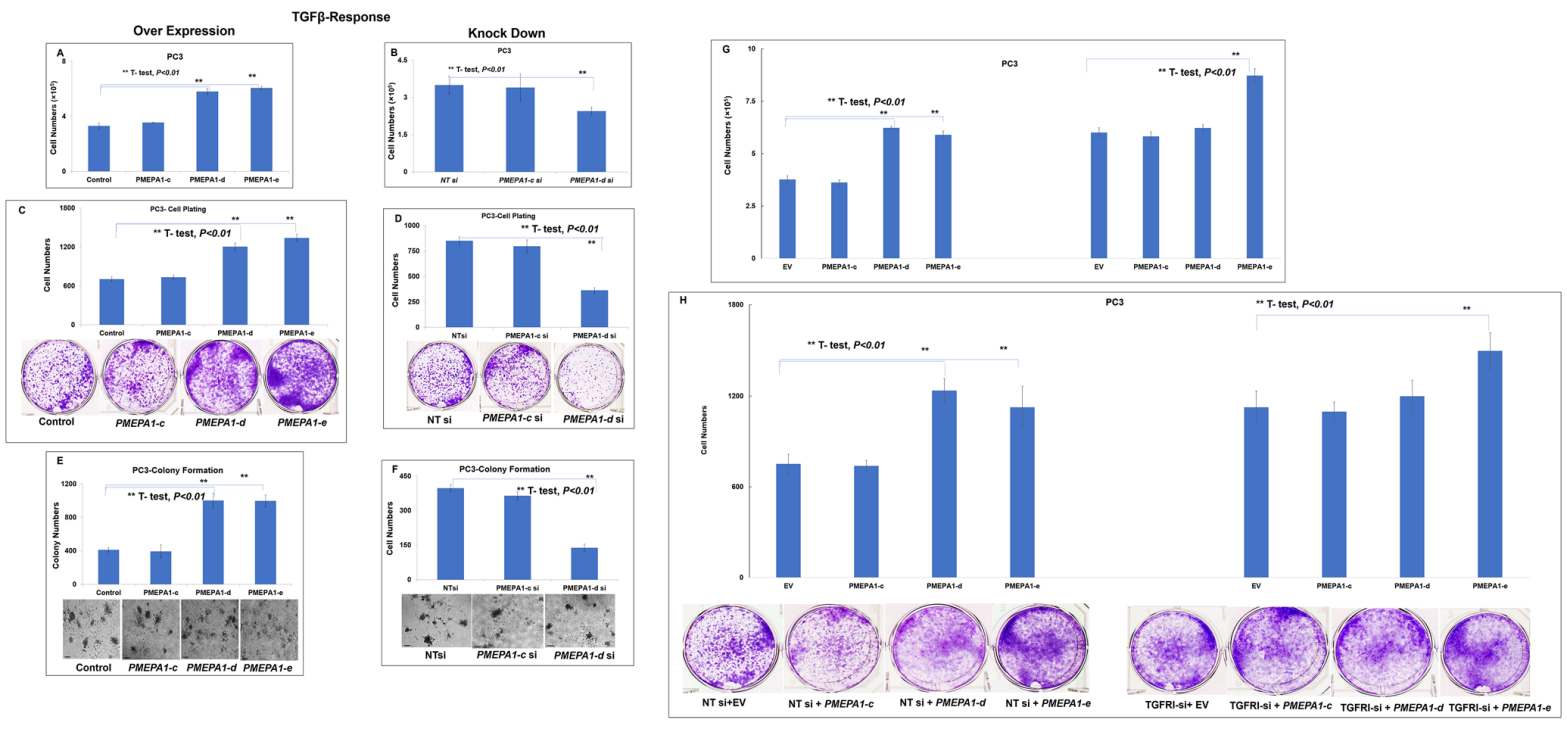
*PMEPA1* isoform *-d* and -*e* promoted the growth of PC3 cells. PC3 cells were transfected with pcDNA3.1-*PMEPA1*-*c*, -*d* and *-e* individually and pcDNA3.1 as control. The assays of cell counting assay (**A**), cell plating efficiency assay (**C**) and colony formation assay in soft agar (**E**) were used to detect the effects of ectopic *PMEPA1* isoforms (*c*, *d* and *e*) on the proliferation of PC3 cells. PC3 cells were transfected with specific siRNA targeting *PMEPA1-c* and *-d* and scramble siRNA as control (The endogenous isoform *e* was not detected in PC3 cells). Similarly, our data revealed effects of knockdown of endogenous *PMEPA-d* on the cell growth rate (**B**), cell plating efficiency (**D**) and soft agar colony formation capacity (**F**) of PC3 cells. Furthermore, the PC3 cells were co-transfected with *TGFRI* siRNA and *PMEPA1* isoforms as indicated. Our data detected the effects of knockdown of endogenous TGFRI on the cell growth promoting effects mediated by *PMEPA1* isoform *a* and *d* in PC3 cells with cell growth assay (**G**) and cell plating efficiency assay (**H**).

Further, we explored the impacts of *PMEPA1* isoform (*c*, *d* and *e*) on the growth of AR/PSA negative but TGF-β signaling positive PC3 cells. The only TGF-β responsive *PMEPA1* isoforms (*c* and *d*) transcripts were detected, and the mRNA level of androgen responsive *PMEPA1-e* isoform was undetectable. Our cell growth assay data indicated that both *PMEPA1-d* and *-e* promoted the growth of PC3 cells (Figure 4A). Consistently, knockdown of *PMEPA1*-*d* resulted in the inhibition of cell growth (Figure 4B). These observations were further supported by the results of cell plating efficiency (Figure 4C and 4D) and soft agar colony formation assays (Figure 4E and 4F). *PMEPA1-c* isoform was not shown to have significant impacts on the cell growth, cell plating efficiency and colony formation capacity in soft agar of PC3 cells. Our study also revealed that overexpression of exogenous *TGFBRI* led to the growth inhibition of the PC3 cells. Moreover, the accelerated cell growth mediated by *PMEPA1-d* was abolished in *TGFRI*-depleted PC3 cells. In contrast, the effects of ectopic *PMEPA1-e* on the cell growth were not affected by *TGFRI* silencing (Figure 4G and 4H). Of note, the colonies formed by PC3 in soft agar were smaller, which made the vision effects of colony formation assay images less sharpened and contrasted. Quantitative image analysis data shown with bar-graph demonstrated that ectopic *PMEPA1-d* and *-e* significantly increased the colony numbers of PC3 cells (*t*-test, *P* < 0.01). Consistently, knockdown of *PMEPA1-d* isoform with siRNA significantly decrease the colony numbers (*t*-test, *P* < 0.01). These findings underscored that the effects of *PMEPA1-d* on cell growth was TGF-β signaling dependent, while *PMEPA1-e* promoted prostate cancer cell growth in TGF-β signaling independent way. Additionally, the growth of both AR positive and negative prostate cancer cells was unaffected by *PMEPA1-c* isoform harboring truncated N-terminal extra-cellular and transmembrane anchoring domains, suggesting that these two domains were essential to maintain cell growth regulating effects of *PMEPA1* isoforms in prostate cancer cells.

### PMEPA1-d isoform inhibited TGF-β signaling in prostate cancer cells

**Figure 5:**
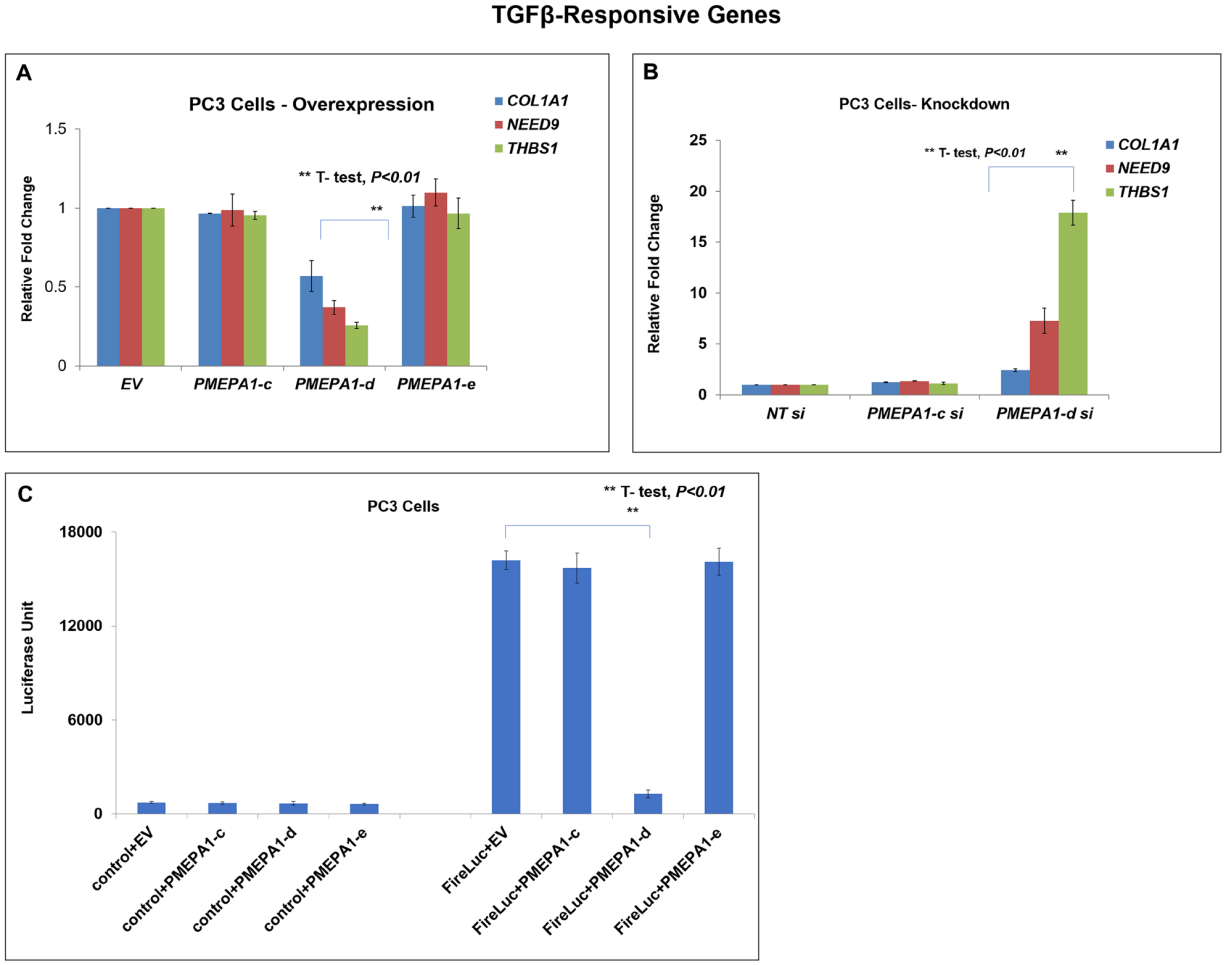
*PMEPA1-d* isoform inhibited TGF-β signaling in PC3 cells. (**A**) PC3 cells were transfected with expression plasmids of *PMEPA1* isoform *c, d* and *e* as indicated. And the transfected cells were harvested 72 hours post transfection. The transcript levels of TGF-β responsive genes *COL1A1, NEDD9* and *THBS1* were detected with Q-PCR assay. (**B**) PC3 cells were transfected with specific siRNA against *PMEPA1* isoform *c, d* and *e*. Q-PCR was used to detect the transcript levels of *COL1A1, NEDD9* and *THBS1*. (**C**) The SMAD luciferase reporter assay was applied to assess the effects of *PMEPA1* isoforms (*c, d* and *e*) on TGF-β signaling in PC3 cells.

*PMEPA1* had been shown to inhibit TGF-β signaling by sequestering R-SMADs [8, 19]. The Smad binding domain involved in TGF-β regulatory feedback loop was localized within the intra-cellular domains of PMEPA1 isoform proteins. Several studies including ours showed that *PMEPA1-a* isoform functioned as TGF-β signaling inhibitor and *PMEPA1-b* had no impact on TGF-β signaling in prostate cancer and other solid tumors. However, whether other *PMEPA1* isoforms (*c*, *d* and *e*) could perturb TGF-β signaling in prostate cancer cells remained unclear. Along these lines, our Q-RT-PCR data showed that ectopic *PMEPA1-d* down-regulated the transcript levels of TGF-β responsive genes *COL1A1*, *NEDD9* and *THBS1* in PC3 cells (Figure 5A). Consistently, knockdown of isoform *d* resulted in increased transcript levels of these TGF-β/SMAD downstream regulated genes (Figure 5B). In contrast, *PMEPA1-c* and *PMEPA1-e* had no impacts on the transcript levels of TGF-β responsive genes. Further, we measured the transcriptional activation of SMADs by co-transfecting PC3 cells with the expression vectors of each *PMEPA1* isoform *c*, *d* and *e* and luciferase reporter vector under the control of SMAD-inducible promoter-enhancer cassette. The luciferase reporter assay revealed robust inhibition of SMAD promoter activity by *PMEPA1-d* in PC3 cells (Figure 5C). On the other hand, overexpression of *PMEPA1-c* or *PMEPA1-e* had no impacts on SMAD transcriptional activation.

### PMEPA1 isoform (d and e) had no impacts on the growth of androgen dependent prostate cancer cells and androgen signaling

**Figure 6:**
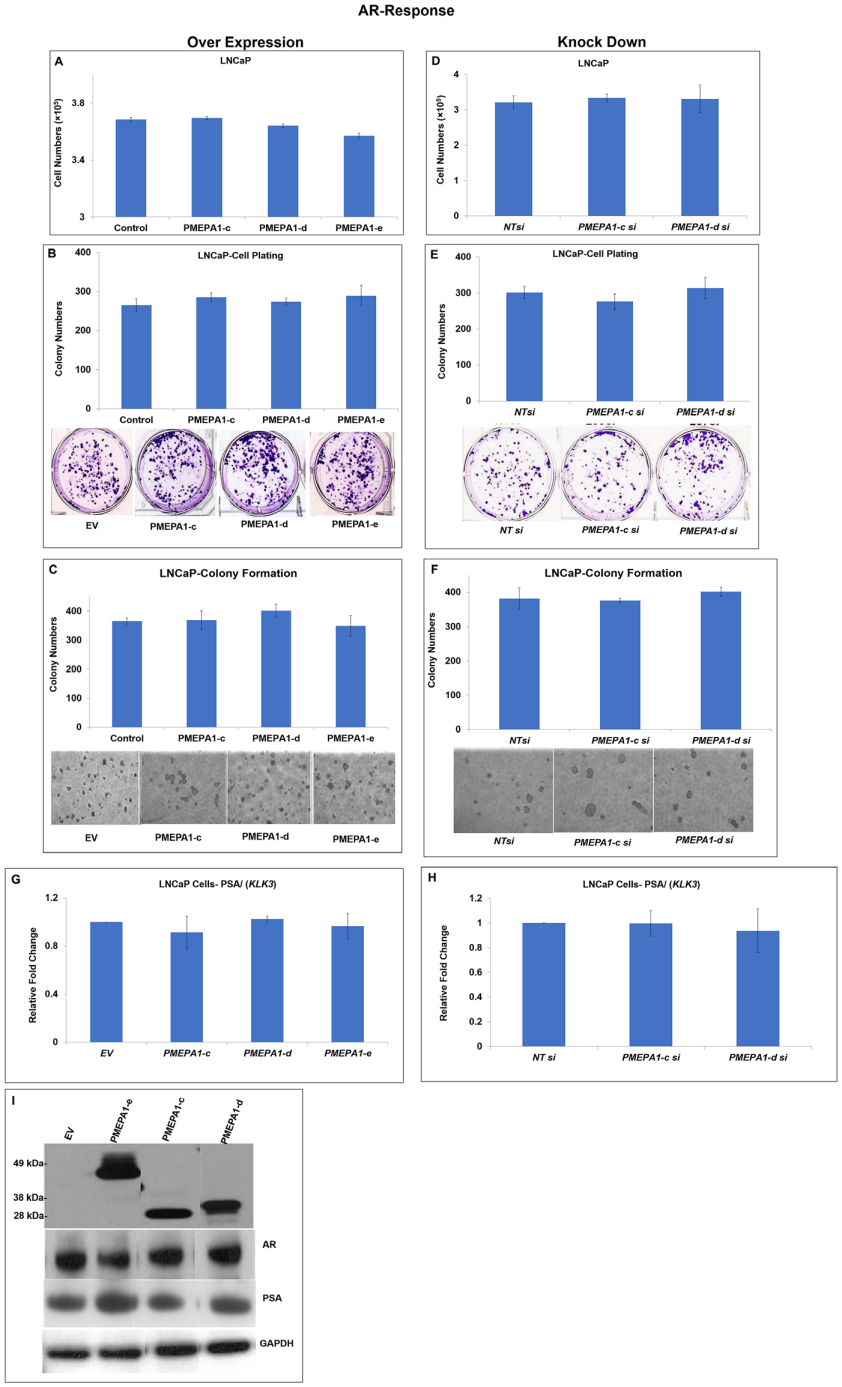
*PMEPA1* isoforms (*c*, *d* and *e*) had no impacts on the cell proliferation and androgen signaling in LNCaP cells. LNCaP cells were transfected with pcDNA3.1-*PMEPA1*-*c*, *-d* and *-e* individually and pcDNA3.1 as control. And the effects of *PMEPA1* isoforms (*c*, *d* and *e*) on the cell proliferation were assessed with (**A**) counting assay, (**B**) cell plating efficiency assay and (**C**) colony formation assay in soft agar. In contrast, LNCaP cells were transfected with specific siRNA targeting *PMEPA1-c* and *-d* and non-target siRNA as control. Similarly, cell growth rate (**D**), cell plating efficiency (**E**) and colony formation capacity in soft agar (**F**) were utilized to study the impacts of knockout of *PMEPA1* isoforms (*c*, *d* and *e*) on cell proliferation of LNCaP cells. In addition, Q-PCR assay was applied to detect the transcript level of PSA (*KLK3*) in LNCaP cells with over-expression of *PMEPA1* isoform *c*, *d* and *e* (**G**) and depletion of *PMEPA1* isoforms (*c* and *d*) (**H**). (**I**) Immunoblotting assay was used to assess the protein levels of PMEPA1 isoforms c, d and e, AR and PSA in LNCaP cells which were transfected with pcDNA3.1-*PMEPA1-c*, *-d* and *-e* individually as indicated and pcDNA3.1 as control. The sizes of PMEPA1 isoform e, d and c were around 49 kDa, 30 kDa and 28 kDa, respectively.

PMEPA1 had also been shown to negatively regulate the protein levels of AR via recruiting E3 ubiquitin ligase NEDD4 in proteasome dependent way through a negative feedback loop [12, 13]. Additionally, it was further revealed that *PMEPA1-b* isoform as an AR signaling inhibitor and *PMEPA1-a* has no impact on AR signaling in prostate cancer cells. Nevertheless, the roles of other *PMEPA1* isoforms (*c*, *d* and *e*) in the context of AR signaling were not fully understood in prostate cancer cells. Towards this, the androgen dependent prostate cancer cell line LNCaP cells were used to assess the impacts of *PMEPA1* isoforms (*c*, *d* and *e*) on androgen signaling and cell growth, cell plating efficiency and colony formation capacity in soft agar. Surprisingly, no *PMEPA1* isoforms was found to have impacts on the growth of androgen responsive LNCaP cells (Figure 6A). Our data further revealed that ectopic *PMEPA1* isoforms (*c*, *d* and *e*) could not alter the cell plating efficiency of LNCaP cells significantly (Figure 6B). The assessment of the anchorage-independent growth capacity in soft agar of LNCaP cells also displayed similar results (Figure 6C). As expected, knockdown of *PMEPA1* isoforms (*c* and *d*) had no impacts on the cell growth, cell plating efficiency and colony formation capacity in soft agar of LNCaP cells (Figure 6D–6F). Along these lines, overexpression and knock down of *PMEPA1-c* and *d* in LNCaP cells were also found to have on impacts on the transcript and protein levels of AR and androgen responsive gene, *KLK3/(*PSA). Moreover, the ectopic *PMEPA1-e* expression had no effects on AR and androgen signaling in LNCaP cells (Figure 6G–6I). The impacts of other PMEPA1 isoforms including isoform a and b on AR and PSA were shown in Supplementary Figure 1. Taken together, our data showed that both AR regulated *PMEPA1* isoform *e* and TGF-β responsive isoforms *c* and *d* could not suppress cell growth of hormone responsive prostate cancer cells and interrupt androgen signaling.

### The decreased mRNA ratios of PMEPA1 isoforms (d and e) indicated higher Gleason score in prostate cancer patients

**Figure 7:**
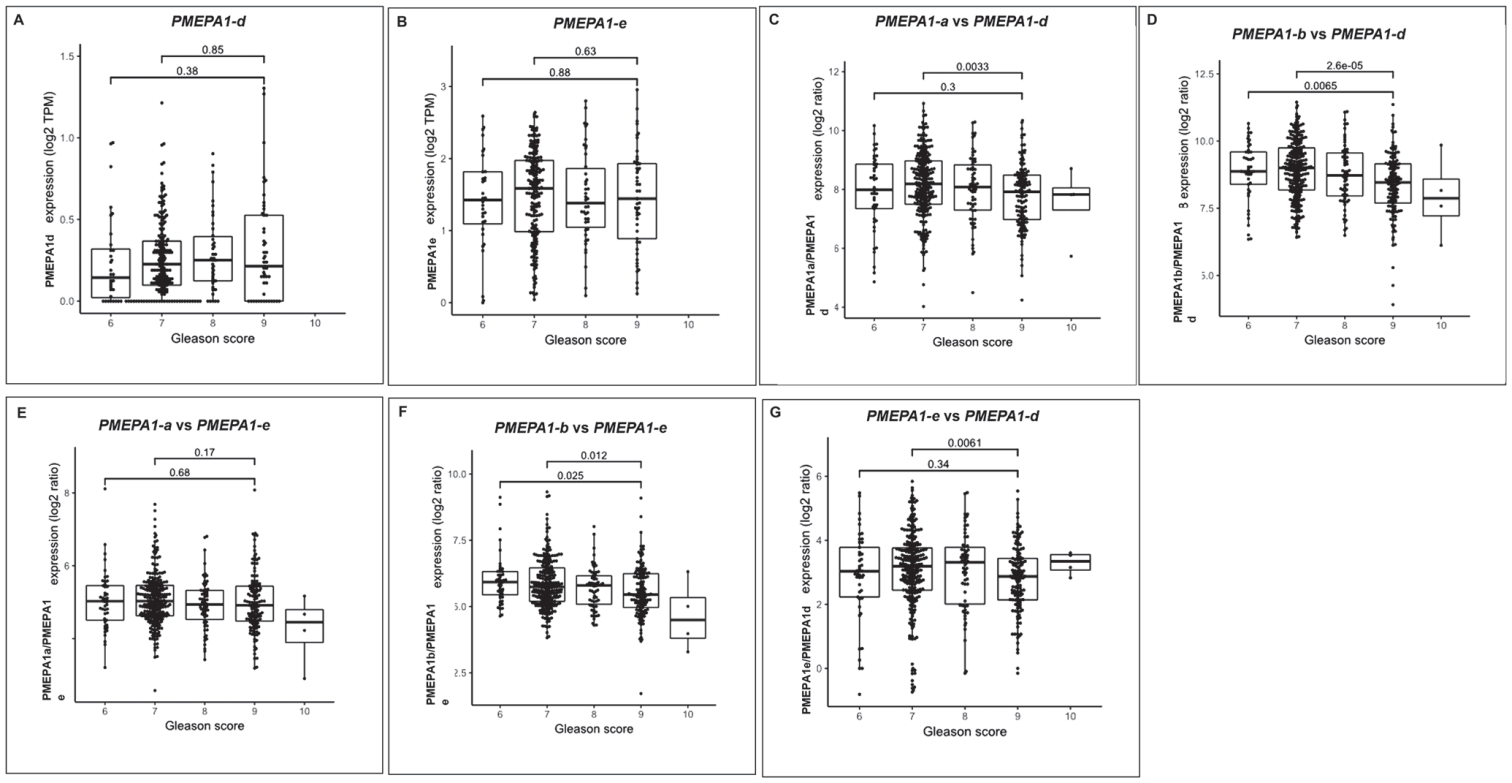
*PMEPA1* isoforms (*d* and *e*) collectively associated with increased Gleason score of prostate cancer. The RNA-sequencing data from TCGA dataset with 499 prostate tumors and unmatched benign prostate tissue was used for analysis the expressions of *PMEPA1* isoforms (*d* and *e*) as well as their associations with Gleason score. The correlations between the transcript levels of individual isoform (*d* or *e*) or the ratio of *PMEPA1* isoforms (*a* vs *d*, *a* vs *e*, *b* vs *d*, *b* vs *e* and *e* vs *d*) and Gleason scores (score 6, 7 and 8 to 10) were analyzed (**A–G**).

Alternative usage of transcript isoforms from the same gene had been hypothesized as an important feature in cancers. One of the aims of our study was to investigate the clinical and molecular characteristics roles of *PMEPA1* isoforms and their expression ratios in prostate tumorigenesis. As a result of the absence of regulation functions on AR/TGF-β signaling of *PMEPA1-c* isoform in prostate cancer cells, we focused on the study of clinical significance and relevance of *PMEPA1* isoforms (*d* and *e*) in prostate cancer patients. We analyzed the TCGA RNA Seq data of unmatched 499 malignant and 50 benign prostate samples. Our analysis showed that the expressions of *PMEPA1* isoforms *d* and *e* were significantly increased in prostate tumor tissue compared to benign tissue (Table 3), consistent with the findings that both *PMEPA1* isoforms (*d* and *e*) promoted the growth of AR negative prostate cancer cells, further suggesting their potential roles in prostate tumorigenesis. Surprisingly, the enhanced transcript level of *PMEPA1-d* or *PMEPA1-e* was not found to be correlated to enhanced Gleason score of prostate cancer individually (Figure 7A–7B). Further, we investigated the transcript ratios between TGF-β associated *PMEPA1* isoforms *(a* and *d*) and AR associated isoforms (*b* and *e*) to measure the alternative splicing ratio as an independent mechanism to control the disease progression. Our findings showed that a decreased ratio of two TGF-β associated *PMEPA1* isoforms (*PMEPA1-a* versus *PMEPA-d*) associated with increased disease Gleason scores (score 7 compared to 8–10, *P* = 0.0035) (Figure 7C). Similarly, the decreased ratio of androgen associated *PMEPA1-b* isoform versus *PMEPA1-d* was strongly correlated to higher Gleason score (score 6 or score 7 compared to score 8–10, *P* = 0.0065 and *P* < 0.01, respectively) (Figure 7D). Further, the expression ratio of *PMEPA1-a* versus *PMEPA1-e* was not associated with Gleason score of prostate cancer (Figure 7E). Whereas, the decreased ratio between transcript levels of *PMEPA-b* and *PMEPA1-e* was highly correlated to increased Gleason score (Gleason score 6 or 7 compared to Gleason score 8–10, *P* = 0.025 and *P* = 0.012, respectively) (Figure 7F). Moreover, the ratio of transcript levels of isoform *e* versus *d* was found to be associated with increased Gleason score (score 7 to 8–10, *P* = 0.0061) (Figure 7G). The further study of the associations between transcript levels of *PMEPA1* isoforms (*d* and *e*) and disease progressions including biochemical recurrence (BCR), metastasis as well as progression free survival rate was warranted.

**Table 3:** Tumor and normal mRNA expression levels of *PMEPA1* isoforms *c*, *d* and *e* in the RNA Seq dataset of TCGA-PRAD v10.0

PMEPA1 Isoforms	Normal Expression Mean Log2 TPM	Tumor Expression Mean Log2 TPM
*PMEPA1-d*	0.1187	0.28735
*PMEPA1-e*	0.9785	1.4356

## DISCUSSION

Through RNA Seq technology for unbiased detection of transcripts, we comprehensively analyzed *PMEPA1* gene isoforms in androgen responsive VCaP, LNCaP prostate cancer cell lines and TCGA dataset of primary prostate tumor tissues. The notable features of the RNA Seq methodologies include the abilities to quantify RNA species including novel non-coding splice variants, RNA at baseline level and a wider coverage of dynamic range of signal [24–26]. Additionally, RNA sequencing technologies had also been applied in biomarker discovery by enabling profiling of alternatively spliced transcripts with high frequency in cancers. Our analysis identified five *PMEPA1* isoforms encoding 287 aa (*PMEPA1-a*), 252 aa (*PMEPA1-b*), 237 aa (*PMEPA1-c*), 259 aa (*PMEPA1-d*) and 344 aa (*PMEPA1-e*). This study focused on one novel isoform *PMEPA1-e* and two less characterized isoforms (*PMEPA1-c*, and *PMEPA1-d*). The amino acid sequence alignment of PMEPA1 revealed the differences among five isoforms only at the N-terminus of protein. On the other hand, both membrane spanning and the C-terminal regions are highly conserved in all the isoforms. The newly identified PMEPA1-e isoform contains a long N-terminal region of 97 aa. As shown in Figure 2A, *PMEPA1* isoforms *a* and *e* were found to utilize the same translation initiation codon. A unique stretch of 57 aa was found in PMEPA1-e isoform in comparison to all other isoforms. Such amino acid sequence variation might be the result of alternative splicing of the exon and/or retention of the intron in the mRNA. The blast analysis of the nucleotide sequence corresponding to unique aa showed that stretch was present in the same locus with a conserved GT and AG at the 5′ and 3′ end, respectively. These features suggested that the long luminal region in PMEPA1-e may be due to the retention of intron. Intron retention was one of the alternative splicing mechanisms requiring both suboptimal 5′ and 3′ splice sites, which was often overlooked or interpreted as splicing mistake where intron was not spliced out [23, 27, 28]. Enhanced levels of retained introns were usually noted in cancer cells, leading to higher diversity in cancer transcriptomes [22]. Minor introns embedded in genes execute functions in signal transduction, cell cycle, DNA damage and information relay [29]. Several tumor suppressors and oncogenes had been reported to possess cancer-related alternative splicing and intron retention [30–35]. Here, we discovered that *PMEPA1*-*e* hosted a partial intron retention between exon 2 and 4. Our findings suggested that alternative splicing events involving *PMEPA1* gene contribute to the transcriptional diversity and may be the basis for the multi-functional attributes of *PMEPA1* gene in prostate cancer.

Our data confirmed the inherent correlations between the expressions of *PMEPA1* isoforms and androgen/TGF-β signaling in prostate cells. Isoform *PMEPA1-e* was androgen responsive, consistent with the observations that *PMEPA1-e* was only detectable in AR positive prostate cancer cells. In contrast, *PMEPA1-c* and *PMEPA1-d* were TGF-β responsive. The *PMEPA1* isoform *c* and *d* were detected in both androgen and TGF-β signaling positive prostate cancer cells although it was only responsive to TGF-β treatment. It has been reported the mutually exclusive expressions of TGF-β and EGF were inhibited by androgen. Then endogenous androgen signaling in hormone dependent prostate cancer cells resulted in the decreased TGF-β and activated EGF signaling, which could ultimately enhance the expression of *PMEPA1* gene [36]. The study of the regulation of *PMEPA1* isoforms expression by EGF signaling was further needed.

It was noted that the cell growth stimulating effects mediated by *PMEPA1-e* was independent of TGF-β signaling in PC3 cells. Furthermore, *PMEPA1-e* had no impacts on the transcript levels of TGF-β responsive genes and SMAD luciferase activity. All these findings explicitly outlined newly discovered *PMEPA1-e* isoform as a gene promoting prostate tumor growth without manipulating classic androgen and TGF-β signaling in prostate tumorigenesis, and the cell growth promoting mechanism of *PMEPA1-e* needed further elucidation. On the other hand, *PMEPA1-d* promoted the growth of AR negative prostate cancer cells in TGF-β signaling dependent way. Consistently, *PMEPA1*-*d* was found to inhibit TGF-β signaling including decreasing the transcript levels of TGF-β responsive genes, as well as suppressing SMAD-dependent reporter activity. Meanwhile, *PMEPA1-d* was not found to promote the growth of androgen responsive LNCaP cells, further highlighting the interactions between intronic TGF-β signaling and *PMEPA1-d*. Of note, *PMEPA1-c* had no impacts on the growth of prostate cancer cells and AR or TGF-β signaling. No extra-cellular was detected in PMEPA1-c protein, and the shorter truncated transmembrane domain was defined in PMEPA1-c protein. In addition, the different protein sequences at N-terminus of PMEPA1-d and -e isoforms were detected. All these findings underlined the importance of N-terminal extracellular domains mediating the distinct functions of *PMEPA1* isoforms.

To identify transcriptomic biomarkers for prostate cancer prognosis, we used TCGA dataset to test the clinical relevance and significance of PMEPA1 isoforms (*d* and *e*). Although dysfunctions of AR/TGF-β signaling had been reported to play critical roles in prostate cancer progression, the transcript levels of individual *PMEPA1* isoforms (*d* and *e*) were not found to associate with disease aggressiveness such as Gleason score in TCGA dataset of human prostate cancer patients. Along these lines, we further investigated the associations between expression ratios of *PMEPA1* isoforms (*b* versus *d* and *e*, as well as *a* versus *d* and *e*) and clinical-pathological features. Our data revealed that the decreased ratios of transcript levels of isoform *b* versus *d* or *e* strongly correlated to higher Gleason score groups (score 6, 7 versus 8–10), which was further supported by the findings that the increased expression levels of *PMEPA1* isoforms *d* and *e* as well as lower transcript level of *PMEPA1-b* in tumor tissue compared to normal prostate. Meanwhile, the similar analysis of TGF-β associated isoforms *PMEPA-a*, and -*d* and androgen regulated isoform *e* revealed the associations between higher Gleason score (score 7 versus 8–10) and increased expression ratios of *PMEPA1* isoforms (isoform *a* versus *d*, isoform *a* versus *e*). Such findings further indicated the interplay between androgen and TGF-β signaling in the disease progression, also the de-regulation of TGF-β signaling tended to happen in later stage of disease. Our results supported the conception that gene isoform expressions as a rich resource for biomarkers predictive of prostate cancer risk stratification. Therefore, the biomarker candidate features of *PMEPA1* isoforms (*d* and *e*) were worthwhile being further validated with different prostate cancer cohorts.

**Figure 8:**
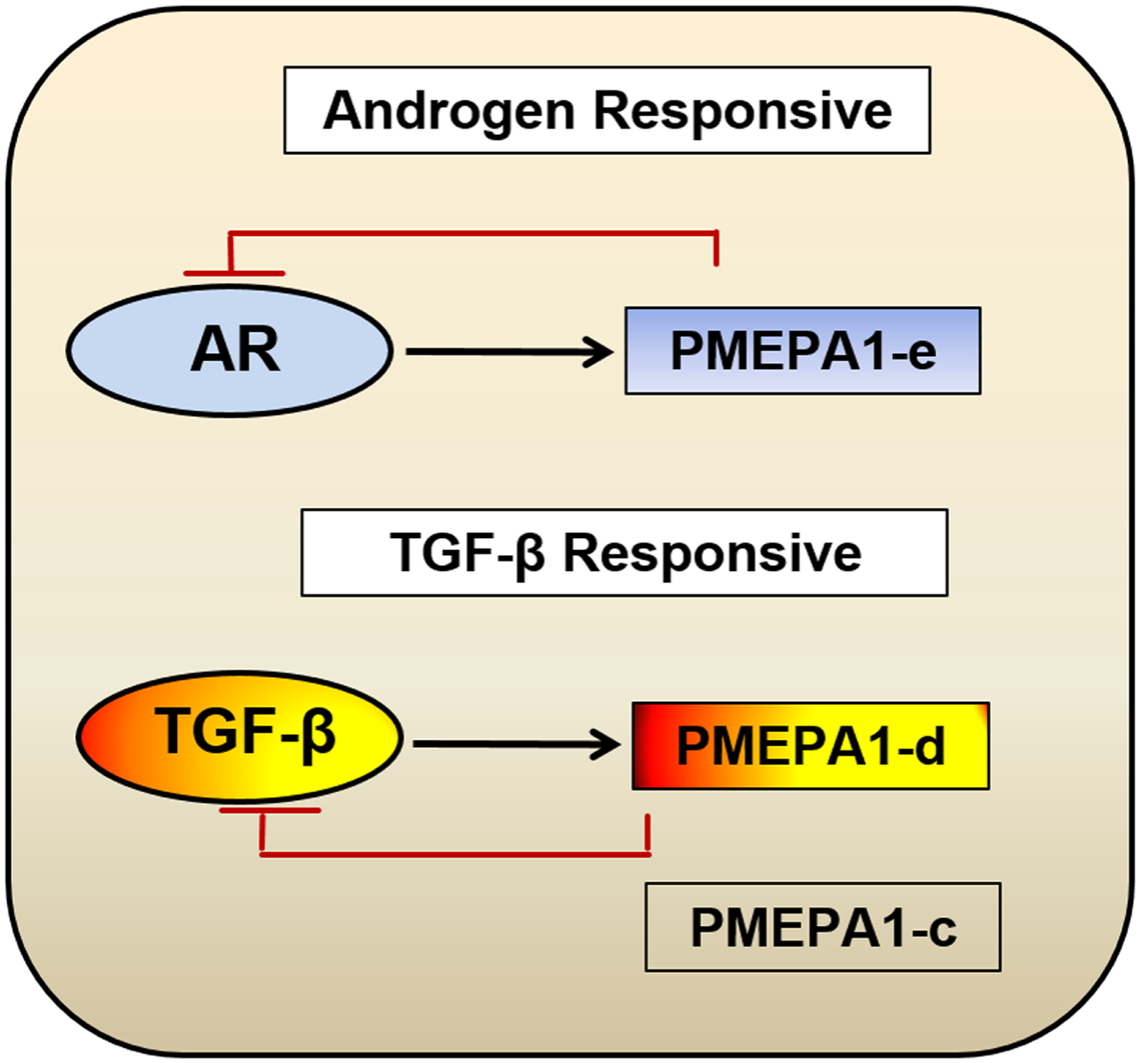
Model for biological function categorization of *PMEPA1* isoforms (*c*, *d* and *e*) in the context of prostate cancer. Our study suggested a model where evaluation of *PMEPA1* isoforms revealed a potentially new mechanism of prostate cancer cell adaptation from androgen dependent to hormone independent, TGF-β controlled cell growth. *PMEPA1-e* were androgen responsive whereas the *PMEPA1* isoform *c* and *d* were TGF-β responsive and only isoform *d* inhibited TGF-β signaling.

Taken together, our study provided new insights into the modulations of AR and TGF-β signaling through understudied isoforms (*d* and *e*) of *PMEPA1* gene, providing the new understandings of gene networks centering AR/TGF-β signaling regulation in the context of prostate cancer. These observations highlighted that both cellular context/*PMEPA1*-isoforms were critical for either AR or TGF-β signaling (Figure 8). Evaluation of *PMEPA1* isoforms *c*, *d* and *e* revealed a potentially new mechanism of prostate cancer cell adaptation from androgen dependent to TGF-β dependent cell growth. Moreover, our study indicated that gene isoform ratio could potentially predict the gene functional consequences and disease progression. Future studies of *PMEPA1* isoforms in prostate and other cancers will greatly benefit the utility of disease prognostic determination and therapeutic stratification.

## MATERIALS AND METHODS

### Cell lines and culture

The cell lines LNCaP, VCaP, PC3 and DU145 were obtained from ATCC and cultured in medium under conditions as the supplier suggested. The LAPC4 cell line was the generous gift from Dr. Charles Sawyers’ lab (University of California at Los Angeles and Memorial Sloan Kettering Cancer Center, New York, NY) and grown in Iscove’s Modified Dulbecco’s Medium with 15% FBS. For androgen and TGF-β treatment experiments, the cells were first pre-treated with 10% charcoal-stripped serum (Gemini Bio-Products, West Sacramento, CA) supplemented medium for 120 hours. Then, the linear dosages of synthetic androgen, R1881 (PerkinElmer, Waltham, MA) (0, 0.1 and 1.0 nM) or human recombinant TGF-β (R&D Systems, Minneapolis, MN) (0, 5.0 and 25.0 ng/mL) was added to the 10% charcoal-stripped serum supplemented fresh medium for 24 hours.

### RNA sequencing

The RNA Seq was carried out on total RNA extracted from VCaP and LNCaP cells. The quality and quantity of RNA were determined by fluorescence-based Qubit RNA HS Assay Kit on Qubit Fluorometer (ThermoFisher Scientific, Waltham, MA). Poly (A)+ RNA was further purified from total RNA samples. Then, the cDNA library was synthesized using TruSeq Stranded mRNA Library Preparation Kit (Illumina, San Diego, CA) per the manufacturer’s protocol. RNA quality indicator (RQI) > 9.0 was used as input for library preparation. Six different libraries were run on a single flow cell lane of an Illumina Hi-Seq 2000 sequencer generating 50 bp paired end reads for VCaP and single end reads for LNCaP cells. PCR based quantification of sequencing libraries were done by KAPA Library Quantification Kit for NGS (Kapa, Wilmington, MA). The raw sequencing data was de-multiplexed (DEMUX) by using bcl2fastq2 Conversion Software 2.17 before alignment. Only quality filtered reads were aligned to the reference human genome (hg19) using TopHat2 [37, 38]. The RNA Seq analysis of TCGA dataset human prostate cancer samples and VCaP, LNCaP cells was used to determine the expression levels of *PMEPA1* isoforms. Fastq files were aligned to the human reference genome (hg19) using Tophat (v2.0.8b) and Bowtie (1.0.1) applying the ensemble gtf option with the GRCh37.59 gtf to build bowtie indexes [37, 38]. Reads were annotated and quantified to a given gene using the Python module HT-SEQ. For gene counts the same ensemble gtf mentioned above was used to provide reference boundaries. The R/Bioconductor package DEXSeq was applied to normalize for library size and perform a variance-stabilizing transformation. Multiple testing was corrected by using the Benjamini-Hochberg procedure to reduce the false discovery rate.

### Plasmids and siRNAs

The pcDNA3.1-HA-*PMEPA1* expression vectors *(PMEPA1* isoform *c*, *d*, and *e*) were generated by GenScript (Piscataway, NJ). The gene was bounded by HindIII and XhoI restriction site at 5′ and 3′, respectively. In addition, pCMV-XL5, pCMV-XL5-AR were described previously (14). The pcDNA3.1-*TGFBR1* plasmid was purchased from GeneScript. Specific siRNA targeting *PMEPA1* isoform *c*, *d*, and *e* and scramble control (D-001810-10-05) were purchased from Dharmacon/Fisher Scientific (Pittsburgh, PA). The siRNA sequences targeting *PMEPA1* isoforms were: *PMEPA1-c* siRNA: 5′-GAACAAGCCTCCTGGTCTTTCTG-3′; *PMEPA1-d* siRNA: 5′-GTGATATACACTCCTTATTTAA-3′ and *PMEPA1-e* siRNA: 5′-CCTGCACGTGCAACTGCAAACGC-3′. The sequence of siRNAs targeting *AR* (GeneID:367) were: the 1:1 mixture of siRNA1: 5′-GCAAAGGTTCTCTGCTAGA-3′ and siRNA2: 5′-TCGAGGCCCTGTAACTTG-3. The sequences of siRNA targeting *TGFBR1*(GeneID:7046) were: the 1:1:1 mixture of siRNA1 5′-GACAUCUAUGCAAUGGGCUUAGUAU-3′, siRNA2 5′-GCAUCUCACUCAUGUUGAUGGUCUA-3′ and siRNA3 5′-AGUAAGACAUGAUUCAGCCACAGAU-3′.

### RNA Isolation and quantitative RT- PCR

RNeasy RNA Isolation Kit (Qiagen, Germantown, MD) was applied to isolate the total RNA from cell pellets. Complementary DNA (cDNA) was synthesized using total RNA (2 µg per reaction) with Omniscript reverse transcriptase and oligo (dT)-12 primers (Qiagen, Germantown, MD). SYBR™ Green PCR Master Mix (Applied Biosystems, Foster City, CA) was used to amplify the targeted genes in cDNA samples on the Stratagene Mx3000P Real-Time PCR system (Agilent Technologies, Santa Clara, CA). *GAPDH* mRNA was used as endogenous control to normalize each sample. Each experiment was performed independently for three times and data were analyzed by using MxPro v.3.2 software (Agilent Technologies, Santa Clara, CA). The changes of transcript levels of genes were determined using the ∆Ct approach. The sequences of Q-PCR primers used for *PMEPA1* isoforms were listed as follows: *PMEPA1-c*: 5′-GGATGAATTCGCTCTGGTCTAG-3′ (forward), 5′-ACCACCATCACCATCATCAC-3′(reverse); *PMEPA1-d*: 5′-ACAGGCGAAAAGTCAAAATGC-3′ (forward), 5′-ACCACCATCACCATCATCAC-3′ (reverse); *PMEPA1-e*: 5′-CTTCCCCGTGTGCAAGAG-3′ (forward), 5′-CTGGATCCTCAGCCACTG-3′ (reverse). The PCR primers for *COL1A1*, *NEDD9*, and *THBS1* [2] and *AR*, PSA (*KLK3*) and *GAPDH* [15] were described previously.

### Cell counting, cell plating efficiency assay and colony formation assay

The cells were seeded into 6 cm culture dishes at the density of 2 × 10^5^ cells/dish. The transfections were mediated by lipofectamine 2000 according to manufacturer’s instructions (Invitrogen, Carlsbad, CA). For cell counting, the cells were treated with trypsin (0.25% trypsin plus EDTA, Life Technology, Carlsbad, CA), collected, re-suspended into 10 ml regular medium, and the single cell suspension was analyzed on hemocytometer for cell counting with trypan blue (Sigma-Aldrich, St. Louis, MO). For cell plating efficiency assay, the colonies were fixed with 4% paraformaldehyde, stained with crystal violet (0.5% w/v), and counted under inverted microscope and evaluated for their survivability. For colony formation assay, each 6-well plate was loaded with 2 ml 0.5% agar as base layer of soft agar (BD Biosciences, San Jose, CA) first, and 2 ml 0.3% agar top layer mixed with the transfected prostate cancer (LNCaP or PC3) cells (0.5 × 10^4^ cells and 5 × 10^4^ cells/ml). The colonies formed in soft agar were counted under inverted microscope after 10 days of transfections. The cellular colonies composed of more than 10 cells were counted as positive colonies.

### SMAD reporter assay

The Cignal SMAD Reporter (luc) Kit (SA Biosciences, Qiagen, Hilden, Germany) was used for the dual luciferase assay. The HEK293 cells were transfected with SMAD reporter, negative control, positive control and PMEPA1 isoforms expression vectors (pcDNA3.1-*PMEPA1-c*, pcDNA3.1-*PMEPA1-d*, and pcDNA3.1-*PMEPA1-e* plasmids) according to manufacturer’s instructions. After 24 hours of transfection, serum free medium was changed to assay medium (Opti-MEM + 0.5% FBS + 0.1 mM NEAA + 1 mM Sodium pyruvate + 100 U/ml penicillin + 100 µg/ml streptomycin) and cells were treated with different doses of human recombinant TGFβ1 (0 and 5.0 ng/mL) for 18 hours. Dual Luciferase assay was performed, and promoter activity values were presented as arbitrary units using a Renilla reporter for internal normalization. Experiments were done in triplicates, and the standard deviations were indicated.

### Analyses of PMEPA1 isoform structure, expression levels and correlation with Gleason scores

The cohort from TCGA database (v10.0) used for analysis of correlations between expressions of *PMEPA1* isoforms and tumor Gleason scores included 499 prostate cancer cases and 50 unmatched normal prostate tissues. The numbers of each Gleason scores group of 499 prostate tumor cases were listed as: 45 cases for score 6, 249 cases for score 7, 64 cases for score 8, 137 cases for score 9, and 4 cases for score 10. Kallisto program was used to estimate the transcript levels of documented *PMEPA1* isoforms (isoform *a*, *b*, *c* and *d*). To identify and assess the mRNA level of unreported isoform, we used the HISAT2 (alignment), StringTie (assembler of RNA Seq alignments into potential transcripts), and Ballgown (annotation) programs. The expression values of *PMEPA1* isoforms in benign and malignant prostate tissues were shown as Log2 Transcripts per Million reads mapped (TPM). The correlations of transcript levels of *PMEPA1-d* or *PMEPA1-e* to Gleason scores were further analyzed. In addition, the expression ratios of *PMEPA1-a* versus *PMEPA1-d*, *PMEPA1-a* versus *PMEPA1-e*, *PMEPA1-b* versus *PMEPA1-d*, *PMEPA1-b* versus *PMEPA1-e*, *PMEPA1-e* versus *PMEPA1-d* were used to analyze the correlations to Gleason scores of prostate cancers.

### Statistical analysis

Significance was calculated using an unpaired *t*-test or ANOVA-test. Two-tailed Student’s *t* test was used to compare between specific groups within a dataset. *P* < 0.05 was considered statistically significant difference. Data were presented as mean ±SEM or +SEM.

## CONCLUSIONS

In summary, this study identified a novel androgen response specific, *PMEPA1-e* isoform and addressed the conundrum of *PMEPA1* driven regulation of androgen or TGF-β signaling. This report provided new insights into differential regulations of AR or TGF-β signaling by different isoforms *PMEPA1-d* and *PMEPA1-e* in prostate cancer. Further, specific isoform ratio provided the new platform for future investigations in deciphering their utility as prognostic markers and therapeutic targets in a given cancer type and/or biological context.

## SUPPLEMENTARY MATERIALS



## Abbreviations

PCa: prostate cancer
PMEPA1: prostate transmembrane protein, androgen induced 1
TCGA: The Cancer Genome Atlas
AR: androgen receptor
TGF-β: Transforming growth factor beta 1
TMEPA1: transmembrane prostate androgen induced protein
STAG1: solid tumor-associated 1 protein
GREF: glucocorticoid responsive element matrix family
GATA: family of transcriptional regulatory proteins
QRT-PCR: quantitative reverse transcription-polymerase chain reaction
ATCC: American Type Culture Collection
ORF: open reading frame
TGFRI: Transforming growth factor beta receptor I
COL1A1: collagen type I alpha 1 chain
NEDD9: Neural Precursor Cell Expressed, Developmentally Down-Regulated 9
THBS1: Thrombospondin 1
KLK3: Kallikrein Related Peptidase 3
PSA: Prostate-specific antigen
BCR: biomechical recurrence
NEDD4: E3 Ubiquitin Protein Ligase NEDD4
RNA Seq: RNA sequencing (Ribonucleic acid sequencing)
mRNA: messenger RNA
EGF: Epidermal growth factor receptor
GAPDH: Glyceraldehyde 3-phosphate dehydrogenase
∆∆Ct: delta delta cycle threshold
cDNA: Complementary DNA
hg19: human reference genome
HT-SEQ: High-throughput sequencing
hTGFb1: human transforming growth factor β1 protein
HISAT2: hierarchical indexing for spliced alignment of transcripts 2
TPM: Transcripts per Million reads mapped
SEM: standard error of the mean
UTR: untranslated region
siRNA: Small interfering RNA

## Notes

**Disclaimer**

The contents of this publication are the sole responsibility of the author(s) and do not necessarily reflect the views, opinions or policies of Uniformed Services University of the Health Sciences (USUHS), The Henry M. Jackson Foundation for the Advancement of Military Medicine, Inc., the Department of Defense (DoD), the Departments of the Army, Navy, or Air Force. Mention of trade names, commercial products, or organizations does not imply endorsement by the U.S. Government.

## References

[1] Siegel RL , Miller KD , Jemal A . Cancer statistics, 2019. CA Cancer J Clin. 2019; 69:7–34. 10.3322/caac.21551. 30620402

[2] Xu LL , Shanmugam N , Segawa T , Sesterhenn IA , McLeod DG , Moul JW , Srivastava S . A novel androgen-regulated gene, PMEPA1, located on chromosome 20q13 exhibits high level expression in prostate. Genomics. 2000; 66:257–63. 10.1006/geno.2000.6214. 10873380

[3] Fournier PG , Juárez P , Jiang G , Clines GA , Niewolna M , Kim HS , Walton HW , Peng XH , Liu Y , Mohammad KS , Wells CD , Chirgwin JM , Guise TA . The TGF-β Signaling Regulator PMEPA1 Suppresses Prostate Cancer Metastases to Bone. Cancer Cell. 2015; 27:809–21. 10.1016/j.ccell.2015.04.009. 25982816 PMC4464909

[4] Yoshikawa T , Sanders AR , Esterling LE , Detera-Wadleigh SD . Multiple transcriptional variants and RNA editing in C18orf1, a novel gene with LDLRA and transmembrane domains on 18p11.2. Genomics. 1998; 47:246–57. 10.1006/geno.1997.5118. 9479497

[5] Nakano N , Maeyama K , Sakata N , Itoh F , Akatsu R , Nakata M , Katsu Y , Ikeno S , Togawa Y , Vo Nguyen TT , Watanabe Y , Kato M , Itoh S . C18 ORF1, a novel negative regulator of transforming growth factor-β signaling. J Biol Chem. 2014; 289:12680–92. 10.1074/jbc.M114.558981. 24627487 PMC4007458

[6] Rae FK , Hooper JD , Nicol DL , Clements JA . Characterization of a novel gene, STAG1/PMEPA1, upregulated in renal cell carcinoma and other solid tumors. Mol Carcinog. 2001; 32:44–53. 10.1002/mc.1063. 11568975

[7] Brunschwig EB , Wilson K , Mack D , Dawson D , Lawrence E , Willson JK , Lu S , Nosrati A , Rerko RM , Swinler S , Beard L , Lutterbaugh JD , Willis J , et al. PMEPA1, a transforming growth factor-beta-induced marker of terminal colonocyte differentiation whose expression is maintained in primary and metastatic colon cancer. Cancer Res. 2003; 63:1568–75. 12670906

[8] Vo Nguyen TT , Watanabe Y , Shiba A , Noguchi M , Itoh S , Kato M . TMEPAI/PMEPA1 enhances tumorigenic activities in lung cancer cells. Cancer Sci. 2014; 105:334–41. 10.1111/cas.12355. 24438557 PMC4317935

[9] Xu LL , Su YP , Labiche R , Segawa T , Shanmugam N , McLeod DG , Moul JW , Srivastava S . Quantitative expression profile of androgen-regulated genes in prostate cancer cells and identification of prostate-specific genes. Int J Cancer. 2001; 92:322–28. 10.1002/ijc.1196. 11291065

[10] Giannini G , Ambrosini MI , Di Marcotullio L , Cerignoli F , Zani M , MacKay AR , Screpanti I , Frati L , Gulino A . EGF- and cell-cycle-regulated STAG1/PMEPA1/ERG1.2 belongs to a conserved gene family and is overexpressed and amplified in breast and ovarian cancer. Mol Carcinog. 2003; 38:188–200. 10.1002/mc.10162. 14639658

[11] Masuda K , Werner T , Maheshwari S , Frisch M , Oh S , Petrovics G , May K , Srikantan V , Srivastava S , Dobi A . Androgen receptor binding sites identified by a GREF_GATA model. J Mol Biol. 2005; 353:763–71. 10.1016/j.jmb.2005.09.009. 16213525

[12] Xu LL , Shi Y , Petrovics G , Sun C , Makarem M , Zhang W , Sesterhenn IA , McLeod DG , Sun L , Moul JW , Srivastava S . PMEPA1, an androgen-regulated NEDD4-binding protein, exhibits cell growth inhibitory function and decreased expression during prostate cancer progression. Cancer Res. 2003; 63:4299–304. 12907594

[13] Li H , Xu LL , Masuda K , Raymundo E , McLeod DG , Dobi A , Srivastava S . A feedback loop between the androgen receptor and a NEDD4-binding protein, PMEPA1, in prostate cancer cells. J Biol Chem. 2008; 283:28988–95. 10.1074/jbc.M710528200. 18703514 PMC2570894

[14] Richter E , Masuda K , Cook C , Ehrich M , Tadese AY , Li H , Owusu A , Srivastava S , Dobi A . A role for DNA methylation in regulating the growth suppressor PMEPA1 gene in prostate cancer. Epigenetics. 2007; 2:100–09. 10.4161/epi.2.2.4611. 18174752

[15] Sharad S , Ravindranath L , Haffner MC , Li H , Yan W , Sesterhenn IA , Chen Y , Ali A , Srinivasan A , McLeod DG , Yegnasubramanian S , Srivastava S , Dobi A , Petrovics G . Methylation of the PMEPA1 gene, a negative regulator of the androgen receptor in prostate cancer. Epigenetics. 2014; 9:918–27. 10.4161/epi.28710. 24694733 PMC4065188

[16] Li H , Mohamed AA , Sharad S , Umeda E , Song Y , Young D , Petrovics G , McLeod DG , Sesterhenn IA , Sreenath T , Dobi A , Srivastava S . Silencing of PMEPA1 accelerates the growth of prostate cancer cells through AR, NEDD4 and PTEN. Oncotarget. 2015; 6:15137–49. 10.18632/oncotarget.3526. 25883222 PMC4558141

[17] Itoh S , Thorikay M , Kowanetz M , Moustakas A , Itoh F , Heldin CH , ten Dijke P . Elucidation of Smad requirement in transforming growth factor-beta type I receptor-induced responses. J Biol Chem. 2003; 278:3751–61. 10.1074/jbc.M208258200. 12446693

[18] Watanabe Y , Itoh S , Goto T , Ohnishi E , Inamitsu M , Itoh F , Satoh K , Wiercinska E , Yang W , Shi L , Tanaka A , Nakano N , Mommaas AM , et al. TMEPAI, a transmembrane TGF-beta-inducible protein, sequesters Smad proteins from active participation in TGF-beta signaling. Mol Cell. 2010; 37:123–34. 10.1016/j.molcel.2009.10.028. 20129061

[19] Singha PK , Pandeswara S , Geng H , Lan R , Venkatachalam MA , Saikumar P . TGF-β induced TMEPAI/PMEPA1 inhibits canonical Smad signaling through R-Smad sequestration and promotes non-canonical PI3K/Akt signaling by reducing PTEN in triple negative breast cancer. Genes Cancer. 2014; 5:320–36. 10.18632/genesandcancer.30. 25352949 PMC4209604

[20] Singha PK , Yeh IT , Venkatachalam MA , Saikumar P . Transforming growth factor-beta (TGF-beta)-inducible gene TMEPAI converts TGF-beta from a tumor suppressor to a tumor promoter in breast cancer. Cancer Res. 2010; 70:6377–83. 10.1158/0008-5472.CAN-10-1180. 20610632 PMC2912953

[21] Liu R , Zhou Z , Huang J , Chen C . PMEPA1 promotes androgen receptor-negative prostate cell proliferation through suppressing the Smad3/4-c-Myc-p21 Cip1 signaling pathway. J Pathol. 2011; 223:683–94. 10.1002/path.2834. 21341270

[22] Dvinge H , Bradley RK . Widespread intron retention diversifies most cancer transcriptomes. Genome Med. 2015; 7:45. 10.1186/s13073-015-0168-9. 26113877 PMC4480902

[23] El Marabti E , Younis I . The Cancer Spliceome: Reprograming of Alternative Splicing in Cancer. Front Mol Biosci. 2018; 5:80. 10.3389/fmolb.2018.00080. 30246013 PMC6137424

[24] Nakano N , Itoh S , Watanabe Y , Maeyama K , Itoh F , Kato M . Requirement of TCF7L2 for TGF-beta-dependent transcriptional activation of the TMEPAI gene. J Biol Chem. 2010; 285:38023–33. 10.1074/jbc.M110.132209. 20889500 PMC2992236

[25] Li W , Dai C , Kang S , Zhou XJ . Integrative analysis of many RNA-seq datasets to study alternative splicing. Methods. 2014; 67:313–24. 10.1016/j.ymeth.2014.02.024. 24583115 PMC4120771

[26] Wang Z , Gerstein M , Snyder M . RNA-Seq: a revolutionary tool for transcriptomics. Nat Rev Genet. 2009; 10:57–63. 10.1038/nrg2484. 19015660 PMC2949280

[27] Goodison S , Yoshida K , Churchman M , Tarin D . Multiple intron retention occurs in tumor cell CD44 mRNA processing. Am J Pathol. 1998; 153:1221–28. 10.1016/S0002-9440(10)65666-0. 9777953 PMC1853036

[28] Michael IP , Kurlender L , Memari N , Yousef GM , Du D , Grass L , Stephan C , Jung K , Diamandis EP . Intron retention: a common splicing event within the human kallikrein gene family. Clin Chem. 2005; 51:506–15. 10.1373/clinchem.2004.042341. 15650036

[29] Turunen JJ , Niemelä EH , Verma B , Frilander MJ . The significant other: splicing by the minor spliceosome. Wiley Interdiscip Rev RNA. 2013; 4:61–76. 10.1002/wrna.1141. 23074130 PMC3584512

[30] Surget S , Khoury MP , Bourdon JC . Uncovering the role of p53 splice variants in human malignancy: a clinical perspective. Onco Targets Ther. 2013; 7:57–68. 10.2147/OTT.S53876. 24379683 PMC3872270

[31] Tejedor JR , Papasaikas P , Valcárcel J . Genome-wide identification of Fas/CD95 alternative splicing regulators reveals links with iron homeostasis. Mol Cell. 2015; 57:23–38. 10.1016/j.molcel.2014.10.029. 25482508

[32] Okumura N , Yoshida H , Kitagishi Y , Nishimura Y , Matsuda S . Alternative splicings on p53, BRCA1 and PTEN genes involved in breast cancer. Biochem Biophys Res Commun. 2011; 413:395–99. 10.1016/j.bbrc.2011.08.098. 21893034

[33] Agrawal S , Eng C . Differential expression of novel naturally occurring splice variants of PTEN and their functional consequences in Cowden syndrome and sporadic breast cancer. Hum Mol Genet. 2006; 15:777–87. 10.1093/hmg/ddi492. 16436456

[34] Vitting-Seerup K , Sandelin A . The Landscape of Isoform Switches in Human Cancers. Mol Cancer Res. 2017; 15:1206–20. 10.1158/1541-7786.MCR-16-0459. 28584021

[35] Pan Q , Shai O , Lee LJ , Frey BJ , Blencowe BJ . Deep surveying of alternative splicing complexity in the human transcriptome by high-throughput sequencing. Nat Genet. 2008; 40:1413–15. 10.1038/ng.259. 18978789

[36] Azami S , Vo Nguyen TT , Watanabe Y , Kato M . Cooperative induction of transmembrane prostate androgen induced protein TMEPAI/PMEPA1 by transforming growth factor-β and epidermal growth factor signaling. Biochem Biophys Res Commun. 2015; 456:580–85. 10.1016/j.bbrc.2014.11.107. 25482449

[37] Kim D , Pertea G , Trapnell C , Pimentel H , Kelley R , Salzberg SL . TopHat2: accurate alignment of transcriptomes in the presence of insertions, deletions and gene fusions. Genome Biol. 2013; 14:R36. 10.1186/gb-2013-14-4-r36. 23618408 PMC4053844

[38] Trapnell C , Roberts A , Goff L , Pertea G , Kim D , Kelley DR , Pimentel H , Salzberg SL , Rinn JL , Pachter L . Differential gene and transcript expression analysis of RNA-seq experiments with TopHat and Cufflinks. Nat Protoc. 2012; 7:562–78. 10.1038/nprot.2012.016. 22383036 PMC3334321

