# Supplementary material for: Characterization of unique PMEPA1 gene splice variants (isoforms d and e) from RNA Seq profiling provides novel insights into prognostic evaluation of prostate cancer
Source: Oncotarget. 2020 Jan 28;11(4):362–77. doi: 10.18632/oncotarget.27406 (PMC6996919; doi:10.18632/oncotarget.27406)
Supplement: Supplementary file 1 [file oncotarget-11-362-s001.pdf]

Characterization of unique *PMEPA1* gene splice variants (isoforms *d* and *e*) from RNA Seq profiling provides novel insights into prognostic evaluation of prostate cancer

SUPPLEMENTARY MATERIALS

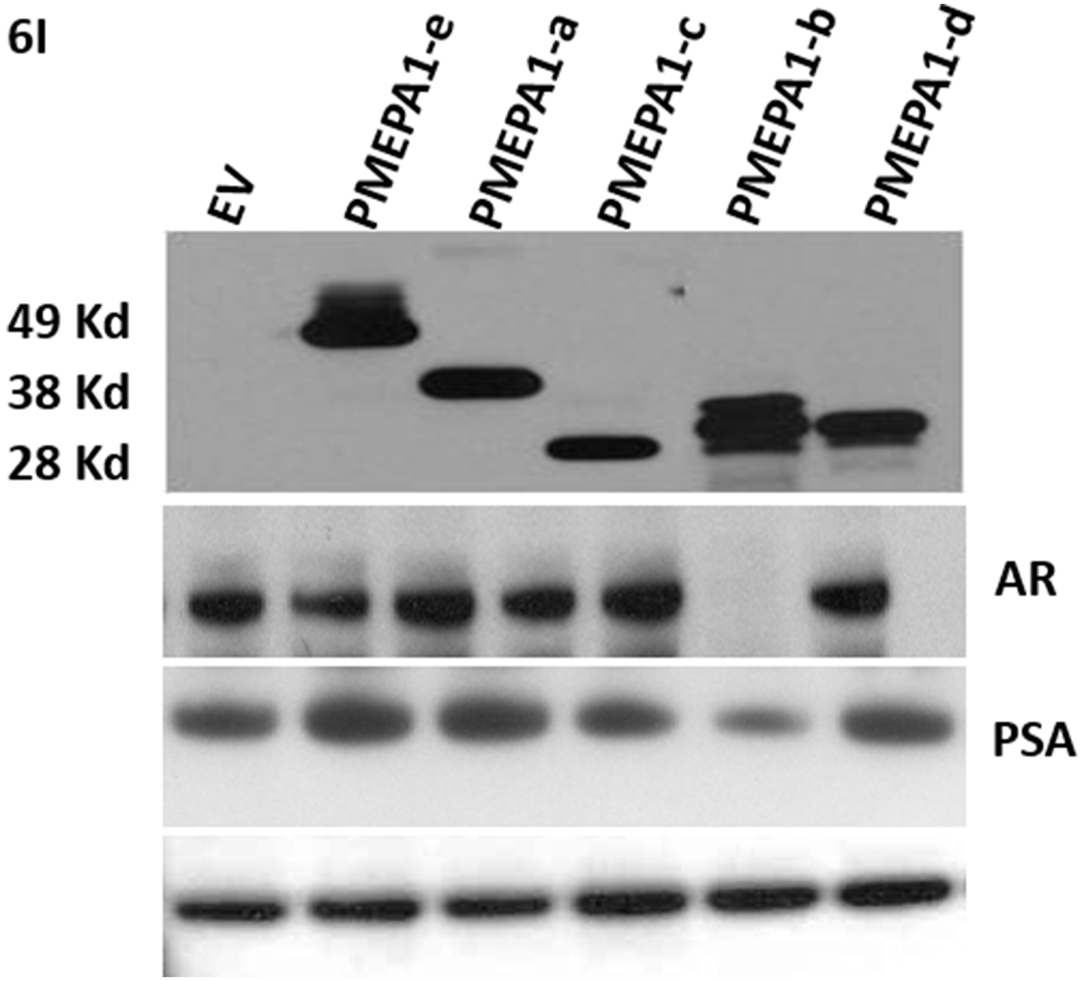

Supplementary Figure 1: Immunoblotting assay was used to assess the protein levels of PMEPA1 isoforms e, a, c, b and d, AR and PSA in LNCaP cells which were transfected with pcDNA3.1-*PMEPA1-e, -a, -c, -b* and *-d* individually as indicated and pcDNA3.1 as control. The sizes of PMEPA1 isoform e, a, c, b and d were around 49, 38, 28, 30 and 30 kDa, respectively.
